# Comparative Mechanical Response of PLA Nanocomposites Reinforced with Multi-Walled Carbon Nanotubes and Halloysite Nanotubes Processed by Injection Moulding

**DOI:** 10.3390/polym17233149

**Published:** 2025-11-27

**Authors:** Christian Cobos, Santiago Ferrándiz, Emilio Rayón, Luis M. López-López, Luis Garzón

**Affiliations:** 1 Grupo de Investigación en Nuevos Materiales y Procesos de Transformación GiMaT, Universidad Politécnica Salesiana, Cuenca 010107, Ecuador; llopez@ups.edu.ec; 2 Instituto Universitario de Investigación en Tecnología de Materiales (IUTM), Universitat Politècnica de València, Plaça Ferrándiz i Carbonell sn, 03801 Alcoi, Alicante, Spain; sferrand@mcm.upv.es (S.F.); emraen@upvnet.upv.es (E.R.); 3 Grupo de Investigación de Física GIF, Universidad Politécnica Salesiana, Cuenca 010107, Ecuador; lgarzon@ups.edu.ec

**Keywords:** polylactic acid (PLA), multi-walled carbon nanotubes (MWCNTs), halloysite nanotubes (HNTs), polymer nanocomposites, mechanical properties, injection moulding, morphological analysis

## Abstract

Polylactic acid (PLA) is a biodegradable polymer with an ever-increasing number of applications, although its inherent brittleness limits its performance somewhat in structural applications. In this study, we analysed the influence of incorporating multi-walled carbon nanotubes (MWCNTs) and halloysite nanotubes (HNTs) at different concentrations (0.5, 0.75 and 1 wt%) on the mechanical properties of injection-moulded PLA nanocomposites. The effects of the nanofillers were characterised by tensile, flexural, and impact tests, hardness measurements, and FESEM examination. The results showed that MWCNTs increased the flexural strength and stiffness by up to 60% compared to neat PLA (84.3 vs. 52.6 MPa), although this was accompanied by a reduction in elongation at break (from 2.30% to 1.57%) due to agglomeration. Conversely, HNTs improved the elongation at break up to 6.39%, enhanced flexural strength by approximately 62% (85.1 MPa), and maintained stiffness around 3.0 GPa, indicating a better balance between strength and ductility. The FESEM micrographs confirmed the presence of clusters in MWCNTs and a more homogeneous dispersion in HNTs, thus explaining the differences in behaviour. Overall, MWCNTs are more suitable for applications requiring high stiffness and strength, whereas HNTs are preferable when greater ductility and impact resistance are required.

## 1. Introduction

Polylactic acid (PLA) is a biodegradable thermoplastic material obtained from natural sources such as corn, wheat, and rice. It can also be used as an alternative to petrochemical derivatives since lactide can be produced on a large scale by microbial fermentation of carbohydrate-rich products [1,2]. PLA has shown promising mechanical properties for applications in the fields of medicine, food, and cosmetics, among others [3]. Despite having excellent biodegradability, non-toxicity, and biocompatibility, it also has certain limitations, such as low strength and hardness, which present challenges in the transformation processes [4]. Research has recently been carried out on obtaining better properties by using nanofillers as reinforcement in the polymeric matrix [5,6,7]; organoclays [8,9,10], carbon nanotubes [11,12], and cellulose nanofibers [13,14,15], e.g., injection moulding technology, used for the mass production of products with complex shapes [16,17,18], and the additive manufacturing technique by 3D printing [19] are among the plastic transformation processes. The former is a cyclic process consisting of four stages (filling, packing, cooling, and ejection) in which the molten material is injected into moulds at high pressure [20,21]. In this technique, the molten mixture of thermoplastic polymer and nanofillers is forced to fill the cavity of the mould used. Injection moulding studies using the PLA-blowing technique are conducted to obtain structural foam [22] at high pressures and temperatures. The thermal and surface properties of PLA/CNT and PLA/MWCNT nanocomposites processed by microinjection moulding have also been investigated [23].

Previous studies analysed these same PLA-based nanocomposites produced by fused filament fabrication (FFF) [24], focusing on their rheological and thermal behaviour. However, the injection moulding process was selected in this work to evaluate the influence of a high-pressure melt processing technique on the mechanical performance of the same materials. Injection moulding allows for the production of standardised test specimens with controlled morphology and density, enabling a direct comparison between additive and conventional manufacturing routes.

In the present study, we analyse the mechanical properties of nanofiller-reinforced PLA samples obtained by injection moulding, including tensile strength, flexural strength, impact resistance, and Shore hardness. The analysed samples were PLA/MWCNT, PLA/HNT, and neat PLA. Based on the intrinsic characteristics of both nanofillers, this study hypothesises that the mechanical behaviour of PLA can be tailored by the type and concentration of nanotubes incorporated: MWCNTs are expected to enhance stiffness and flexural strength, while HNTs are anticipated to improve ductility and impact resistance. The research aims to verify this hypothesis by comparing the mechanical response and fracture morphology of both systems under identical processing conditions.

## 2. Materials and Methods

### 2.1. Sample Preparation

The materials used in this study were neat PLA and PLA loaded with MWCNTs and HNTs at 0.5, 0.75, and 1% by weight. Nanofiller contents of 0.5, 0.75, and 1.0 wt% were selected based on preliminary tests and literature reports [25]. This concentration range allows for evaluating the reinforcement effects of MWCNTs and HNTs while preserving good melt flow, homogeneous mixing, and injection stability. Prior to obtaining the PLA polymer matrix nanocomposites, the polymer was subjected to a drying process at 60 °C for 8 h [26]. PLA and nanofillers were melt-compounded in a co-rotating twin-screw extruder to achieve homogeneous dispersion. The PLA/MWCNTs and PLA/HNT nanocomposites were obtained by direct melt mixing of the materials in a co-rotating twin-screw extruder (DUPRA, Alicante, Spain) with 30 mm diameter extrusion screws, an L/D ratio of 20, a speed of 40 rpm, and a temperature profile between 200 and 205 °C.

**Figure 1:**
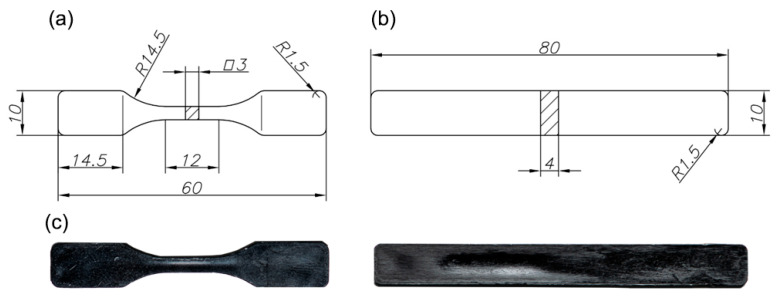
Schematic diagram of the (**a**) tensile specimens and (**b**) flexural and impact specimens; (**c**) photographs of the obtained samples. All measurements are in mm.

After compounding and pelletizing the nanocomposites, test specimens were injection moulded using a Babyplast 610P machine (BETAPLAST, Madrid, Spain) with a barrel temperature profile of 185/205/215 °C. Tensile and flexural samples were prepared according to ASTM D638 [27] and ASTM D790 [28], respectively. Figure 1 shows the obtained samples for tensile, flexural, and impact testing.

For each formulation, 9 specimens were tested for each mechanical property evaluated. To ensure data consistency, the highest and lowest values from each dataset were discarded, and the mean of the remaining seven measurements was reported as the representative value for each composition.

### 2.2. Tensile and Flexural Tests

Tensile and flexural tests were carried out using a Universal Testing Machine (Instron, model 5967, Buckinghamshire, Milton Keynes, UK) equipped with a 30 kN load cell. Tensile tests followed ISO 527-2 [29] with a 20 N preload, a crosshead speed of 2 mm/min, and a grip separation of 30 mm [30]. Three-point bending tests were conducted in accordance with ISO 14125 [31] with a support span of 50 mm and a crosshead speed of 5 mm/min.

### 2.3. Impact Testing

The impact strength of unnotched samples was measured at room temperature using an Izod impact tester (Model LY-X-JUD5.5) supplied by Dongguan Liyi Environmental Technology Co., Ltd., Dongguan, China. The tester was equipped with a pendulum of 11 J nominal energy. Five impact tests were performed according to the ASTM D256 [32] standard, and the mean value was reported for each composition. The absorbed energy was normalised by the cross-sectional area of the specimens and expressed in kJ/m^2^, which allows for comparison among formulations and explains the higher absolute values obtained relative to tests performed with 4 J pendulums.

### 2.4. Hardness Testing

Hardness of PLA/MWCNTs and PLA/HNTs nanocomposites was measured using a Shore-A and Shore-D hardness tester (Bot Instruments, model 673D, Alicante, Spain) according to ISO 868:2003 [33].

### 2.5. Field Emission Scanning Electron Microscope (FESEM)

Fracture morphology and nanofiller distribution in the polymer matrix were examined on fractured cross-section samples using a Field Emission Scanning Electron Microscope (FESEM, Zeiss Auriga, Oxford Instruments, Wiesbaden, Germany), equipped with a secondary electron (SE) and a backscattered electron (BSE) detector.

## 3. Results

### 3.1. Hardness

Neat PLA exhibited Shore A and Shore D hardness values of 73.4 and 82.8, respectively. However, the hardness results were not significantly affected by the nanofiller weight fractions investigated, with a variation of only ±4 hardness units on both scales. This may be due to the injection process requiring high pressures to fill the cavities, thus homogenising the material when filling the specimens. Furthermore, the similarity in hardness between neat PLA and the nanocomposites is due to the fact that they are compounds with a low amount of filler. What really influences the resistance to indenter penetration is the polymer itself, which explains the similarity we found in hardness.

### 3.2. Tensile and Flexural Results

The influence of MWCNT and HNT nanofillers was analysed at 0, 0.5, 0.75, and 1 wt% concentrations. Table 1 summarises the obtained results.

The results obtained for neat PLA revealed a tensile strength of 64.79 MPa and an elastic modulus of 4.35 GPa; these values were within the range reported in the literature for this polymer. However, its deformation at break was limited (2.30%), confirming the brittle nature of the material and its poor capacity to absorb deformation before fracture. These results served as the reference for evaluating the effect of nanofiller incorporation. When 0.5 wt% carbon nanotubes (MWCNTs) were added to PLA, tensile strength slightly increased (66.11 MPa). However, at higher loadings (0.75 and 1.0 wt%), both tensile strength and elongation decreased compared with neat PLA. The formulations containing halloysite nanotubes (HNTs) exhibited a tensile strength that increased slightly with nanofiller content, reaching a maximum of 67.01 MPa at 1 wt%, while strain at break increased significantly to 6.39% at 0.5 wt%. Therefore, HNTs not only contribute to maintaining the material’s strength but also improve its ductility and toughness.

The formulations in this study were also tested under flexural loading. Table 2 summarises the average results of bending tests.

Flexural results reveal that incorporating MWCNTs significantly increased matrix strength and stiffness: the maximum flexural strength was reached at 0.75 wt% (84.32 MPa), approximately 60% higher than that for neat PLA, while the flexural modulus increased from 3.00 to 3.36 GPa. However, at 1 wt% loading, a slight decrease in strength and deformation was found, possibly due to particle agglomeration. Moreover, nanocomposites with HNTs mainly improved ductility, reaching a maximum deformation of 4.84 at 0.5 wt%, while the strength increased to 85.12 MPa at 0.75 wt%. The flexural modulus remained close to 3.0 GPa.

### 3.3. Impact Strength

Impact behaviour was examined across the formulations to quantify the reinforcement effect on absorbed energy. The obtained results are summarised in Table 3.

The results show that the additions of 0.5–0.75% wt% MWCNTs reduce the impact strength, consistent with the increased stiffness and lower toughness reported under tensile deformations, whereas a 1 wt% loading leads to higher absorbed impact energy. At low HNT loadings (0.5–0.75 wt%), the absorbed impact energy exceeds that of neat PLA, consistent with improved toughness, whereas at 1 wt% it decreases slightly.

The differences in mechanical behaviour observed under deformation and impact stresses as a function of nanofiller type and concentration can be rationalised in terms of (i) miscibility and coherence between the nanofiller and PLA; (ii) the ability of the nanofiller either to disperse or to form agglomerates within the matrix phase; and (iii) the intrinsic nature of the nanofiller and its own mechanical response. To examine these hypotheses, a detailed microstructural study of the state of the nanofillers in PLA was carried out by electron microscopy, and the corresponding results are discussed below.

### 3.4. Field Emission Scanning Electron Microscope (FESEM)

**Figure 2:**
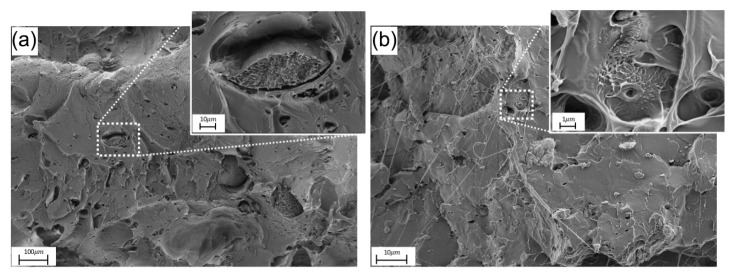
FESEM micrographs of the fracture surface of the PLA/MWCNT nanocomposite (**a**) nanotube agglomerates; (**b**) zones with embedded nanotubes.

In order to determine how the nanofiller is distributed within the matrix phase, as well as the nature of the interaction between both phases, an FESEM study was carried out on the fractured sections of all formulations. For clarity and conciseness, only the most representative images at 0.75 wt% are shown. Figure 2 shows FESEM micrographs of the fracture surfaces of neat PLA, PLA/MWCNT at 0.75 wt%.

FESEM micrographs of the PLA/MWCNT nanocomposite reveal a predominantly brittle fracture surface, characterised by voids, sharp facets, and only limited plastic deformation, as shown in Figure 2a. The inset image shows carbon nanotubes arranged in agglomerates (clusters), indicative of poor dispersion within the matrix. These agglomerates exhibit weak interfacial adhesion to the surrounding PLA, as suggested by the presence of interfacial gaps. The density and size of these agglomerated grains become more evident as the nanofiller content increases, particularly at 0.75 wt% and 1 wt%. Poor cohesion between the agglomerated nanofiller particles and the polymer matrix lowers the effective density of the material and promotes local stress concentration under loading. Because the volumetric density of agglomerates increases at 0.75 wt% and 1 wt%, this behaviour explains the drop in tensile strength and strain at break observed in the mechanical characterisation. Nevertheless, localised regions containing well-dispersed nanotubes with adequate interfacial adhesion were also identified within the PLA matrix at all MWCNT concentrations (Figure 2b), supporting the viability of this nanofiller for PLA and justifying the increase in mechanical strength observed up to 0.5 wt% MWCNTs. These observations suggest that optimising the melt-mixing/compounding conditions to reduce weakly cohesive agglomerates would improve the mechanical response of these formulations.

**Figure 3:**
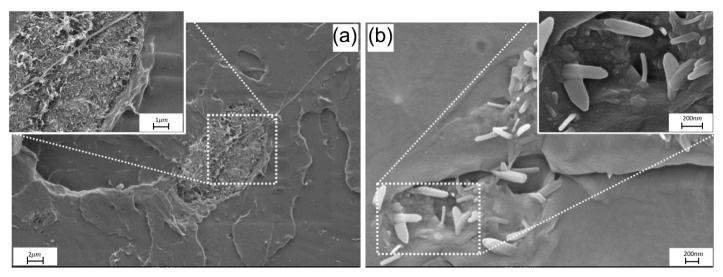
FESEM micrographs of the fracture surface of the PLA/HNT nanocomposite: (**a**) halloysite nanotube agglomerates; (**b**) embedded nanotubes and evidence of pull-out from the matrix.

For HNT-filled formulations, brittle fracture behaviour and agglomeration of the nanofiller into small clusters dispersed within the matrix phase were also observed (Figure 3). However, the agglomerate–matrix interface showed good interfacial cohesion. The superior interfacial compatibility observed for the HNT-filled formulations, compared with the MWCNT-based systems, is consistent with the higher flexural strain and, on average across the concentration range, the higher impact strength.

## 4. Discussion

**Figure 4:**
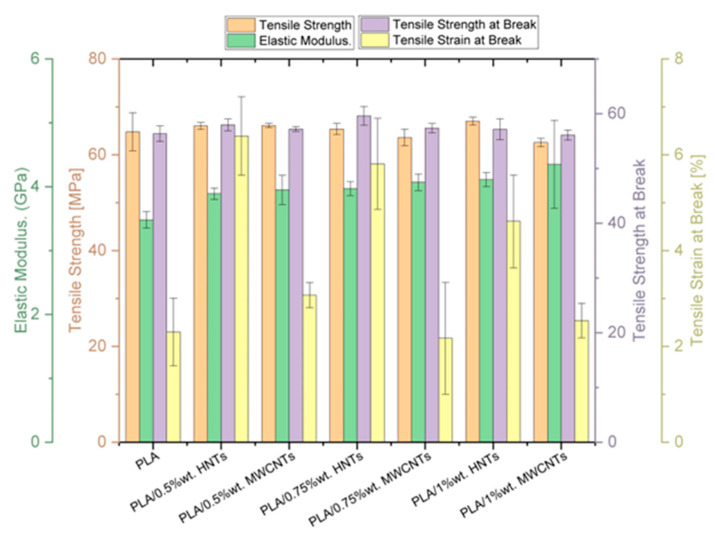
Tensile results for all the PLA/MWCNTs and PLA/HNTs formulations.

**Figure 5:**
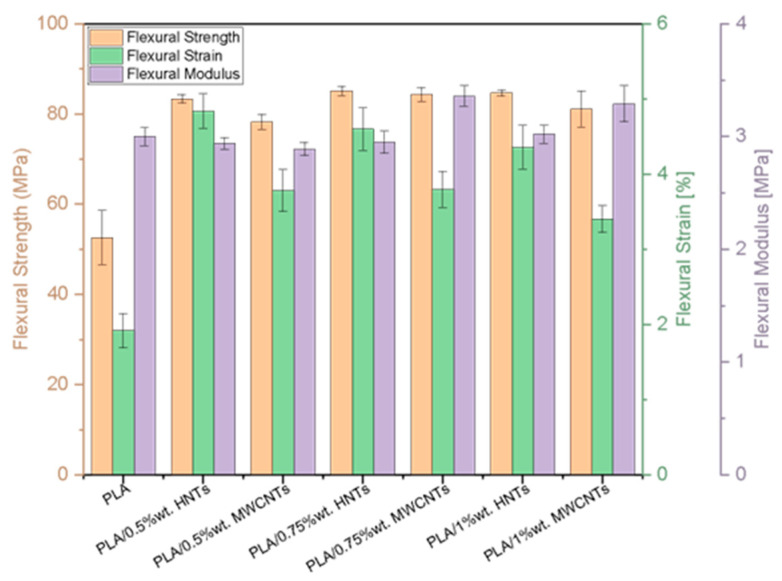
Comparison of results of nanocomposite bending tests.

To support the discussion, the mechanical results obtained for all formulations of this study are represented in Figure 4 and Figure 5.

Hardness of neat PLA was not significantly affected by the incorporation of MWCNTs or HNTs at the concentrations investigated. The low effectiveness of these nanofillers as hardening agents could be explained by the fact that their content is not sufficiently high to produce a detectable effect in indentation hardness measurements, since this technique probes only a very small material volume and does not capture deformation-related hardening mechanisms. Therefore, Shore hardness was not an appropriate technique for evaluating the reinforcement effects at low nanoparticle concentrations.

The tensile results show that adding a low concentration of MWCNTs (0.5 wt%) yields a slight increase in tensile strength, whereas higher contents reduce both tensile strength and strain at break. This behaviour is attributed to nanotube agglomeration and poor interfacial cohesion within the matrix, which limit reinforcement efficiency. By contrast, incorporating HNTs maintained—or even increased—tensile strength while significantly increasing strain at break, indicating a beneficial effect on ductility. Overall, the reinforcement response depends not only on the intrinsic properties of the nanofillers but also on their dispersion state and the quality of interfacial adhesion to the PLA.

In the flexural tests, MWCNTs proved to be more effective at increasing stiffness and strength, reaching their maximum values at 0.75 wt%, although with a decrease at higher contents due to a similar agglomeration effect as observed in tensile testing. The HNTs contributed more significantly to ductility, maintaining strength and modulus at levels similar to pure PLA, suggesting that their contribution is aimed at improving deformation capacity without an appreciable loss of stiffness. Overall, the flexural results demonstrated complementary behaviour: MWCNTs reinforce structural stiffness, while HNTs promote greater damage tolerance, as can be seen in Figure 5.

Impact tests confirmed the different nature of both reinforcements; at low concentrations, MWCNTs reduced the absorbed energy, indicating greater fragility due to their stiffening effect; however, partial recovery of the absorbed energy was found at 1 wt%, possibly associated with the formation of more efficient networks for load transfer. In contrast, HNTs increased impact absorption at intermediate concentrations, thanks to their tubular morphology and greater dispersion in the matrix, favouring energy dissipation. These results suggest that HNTs are more appropriate for applications requiring improved PLA toughness, while MWCNTs are more effective when structural stiffness is the priority.

FESEM analysis confirmed that both MWCNTs and HNTs are viable nanofillers for PLA processed by injection moulding, and that their incorporation influences the mechanical response of the resulting nanocomposites. In both cases, the nanofillers are able to appear coherently embedded within the PLA matrix; however, when the concentration reached 0.75 wt% and above, agglomerated nanofiller domains were detected. For HNTs, these agglomerates still showed good compatibility with the PLA phase, whereas MWCNT agglomerates tended to debond from the matrix, thereby rationalising the loss of mechanical performance, particularly the reduction in strain at break and impact strength.

In future studies, it will be necessary to determine whether these aggregates arise from a saturation limit of PLA in terms of the maximum nanofiller content that can be effectively dispersed, or whether, alternatively, agglomerates are intrinsically likely to form at any concentration, and increasing nanofiller loading simply raises the probability of encountering these unwanted domains. In any case, further work could focus on identifying the origin of this agglomerated phase and on exploring compatibilisers, surfactants, or modified processing conditions to improve MWCNT cohesion and, where possible, further enhance HNT dispersion.

## 5. Conclusions

This study demonstrated that incorporating MWCNTs and HNTs into injection-moulded PLA induces effects in its mechanical response: MWCNTs increase stiffness and flexural strength, although with limitations in ductility and impact due to agglomeration and interfacial adhesion problems, while HNTs significantly improve both toughness and deformation at break, maintaining strength values comparable to neat PLA. The FESEM analysis confirmed that both reinforcements exhibit brittle fracture and agglomeration of the nanofillers, but with greater homogeneity in the HNTs. In terms of applications, MWCNTs are suitable for components requiring high stiffness, while HNTs offer a more favourable alternative for parts demanding greater energy absorption and damage tolerance, highlighting the importance of selecting the appropriate nanofiller and concentration according to the type of performance required by the application.

## Figures and Tables

**Table 1:** Results of tensile tests of pure PLA and PLA reinforced with nanofillers.

Nanocomposites	Tensile Testing				
	Tensile Strength [MPa]	Tensile Strain at Tensile Strength [%]	Elastic Modulus [GPa]	Tensile Strength at Break [MPa]	Tensile Strain at Break [%]
Neat PLA	64.79 ± 3.96	1.49 ± 0.22	4.35 ± 0.69	59.60 ± 1.72	2.30 ± 0.71
PLA 0.5 wt% MWCNTs	66.11 ± 0.45	1.70 ± 0.04	3.89 ± 0.09	57.37 ± 0.85	3.07 ± 0.26
PLA 0.75 wt% MWCNTs	63.60 ± 1.73	1.61 ± 0.08	3.95 ± 0.23	57.16 ± 1.91	2.17 ± 1.17
PLA 1 wt% MWCNTs	62.56 ± 0.89	1.57 ± 0.03	3.97 ± 0.11	56.35 ± 1.41	2.54 ± 0.36
PLA 0.5 wt% HNTs	66.01 ± 0.73	1.61 ± 0.04	4.11 ± 0.11	57.19 ± 0.44	6.39 ± 0.82
PLA 0.75 wt% HNTs	65.37 ± 1.19	1.88 ± 0.06	3.48 ± 0.13	56.12 ± 0.92	5.81 ± 0.95
PLA 1 wt% HNTs	67.01 ± 0.81	1.65 ± 0.05	4.07 ± 0.13	57.96 ± 1.07	4.61 ± 0.97

**Table 3:** Summary of impact energy, absorbed energy, and intensity of the samples tested by Izod.

	Neat PLA	PLA/wt% MWCNTs			PLA/wt% HNTs		
		0.5%	0.75%	1%	0.5%	0.75%	1%
Impact energy (J)	6.42	4.26	4.01	6.76	5.43	5.89	5.31
Impact strength (kJ/m^2^)	160.52	106.62	100.47	169.04	135.77	147.30	123.76

**Table 2:** Bending test results for neat PLA and nanofiller-reinforced PLA.

Nanocomposites	Flexural Testing		
	Flexural Strength [MPa]	Flexural Strain [%]	Flexural Modulus [MPa]
Neat PLA	52.64 ± 5.95	1.92 ± 0.23	3.00 ± 0.08
PLA 0.5 wt% MWCNTs	78.24 ± 1.69	3.78 ± 0.28	2.89 ± 0.06
PLA 0.75 wt% MWCNTs	84.32 ± 1.57	3.80 ± 0.24	3.36 ± 0.09
PLA 1 wt% MWCNTs	81.08 ± 3.99	3.40 ± 0.18	3.29 ± 0.16
PLA 0.5 wt% HNTs	83.34 ± 1.00	4.84 ± 0.23	2.94 ± 0.05
PLA 0.75 wt% HNTs	85.12 ± 1.01	4.60 ± 0.28	2.95 ± 0.10
PLA 1 wt% HNTs	84.70 ± 0.73	4.36 ± 0.29	3.02 ± 0.08

## Data Availability

The raw data supporting the conclusions of this article will be made available by the authors on request.
